# Liquid–liquid phase separation of tau: From molecular biophysics to physiology and disease

**DOI:** 10.1002/pro.4093

**Published:** 2021-05-14

**Authors:** Sandeep K. Rai, Adriana Savastano, Priyanka Singh, Samrat Mukhopadhyay, Markus Zweckstetter

**Affiliations:** ^1^ Centre for Protein Science, Design and Engineering, Department of Biological Sciences, and Department of Chemical Sciences Indian Institute of Science Education and Research (IISER) Mohali India; ^2^ Research group Translational Structural Biology German Center for Neurodegenerative Diseases (DZNE) Göttingen Germany; ^3^ Department for NMR‐based Structural Biology Max Planck Institute for Biophysical Chemistry Göttingen Germany

## Abstract

Biomolecular condensation via liquid–liquid phase separation (LLPS) of intrinsically disordered proteins/regions (IDPs/IDRs), with and without nucleic acids, has drawn widespread interest due to the rapidly unfolding role of phase‐separated condensates in a diverse range of cellular functions and human diseases. Biomolecular condensates form via transient and multivalent intermolecular forces that sequester proteins and nucleic acids into liquid‐like membrane‐less compartments. However, aberrant phase transitions into gel‐like or solid‐like aggregates might play an important role in neurodegenerative and other diseases. Tau, a microtubule‐associated neuronal IDP, is involved in microtubule stabilization, regulates axonal outgrowth and transport in neurons. A growing body of evidence indicates that tau can accomplish some of its cellular activities via LLPS. However, liquid‐to‐solid transition resulting in the abnormal aggregation of tau is associated with neurodegenerative diseases. The physical chemistry of tau is crucial for governing its propensity for biomolecular condensation which is governed by various intermolecular and intramolecular interactions leading to simple one‐component and complex multi‐component condensates. In this review, we aim at capturing the current scientific state in unveiling the intriguing molecular mechanism of phase separation of tau. We particularly focus on the amalgamation of existing and emerging biophysical tools that offer unique spatiotemporal resolutions on a wide range of length‐ and time‐scales. We also discuss the link between quantitative biophysical measurements and novel biological insights into biomolecular condensation of tau. We believe that this account will provide a broad and multidisciplinary view of phase separation of tau and its association with physiology and disease.

## 1 TAU IN PHYSIOLOGY AND PATHOLOGY

### 1.1 Identification and properties of tau

**FIGURE 1:**
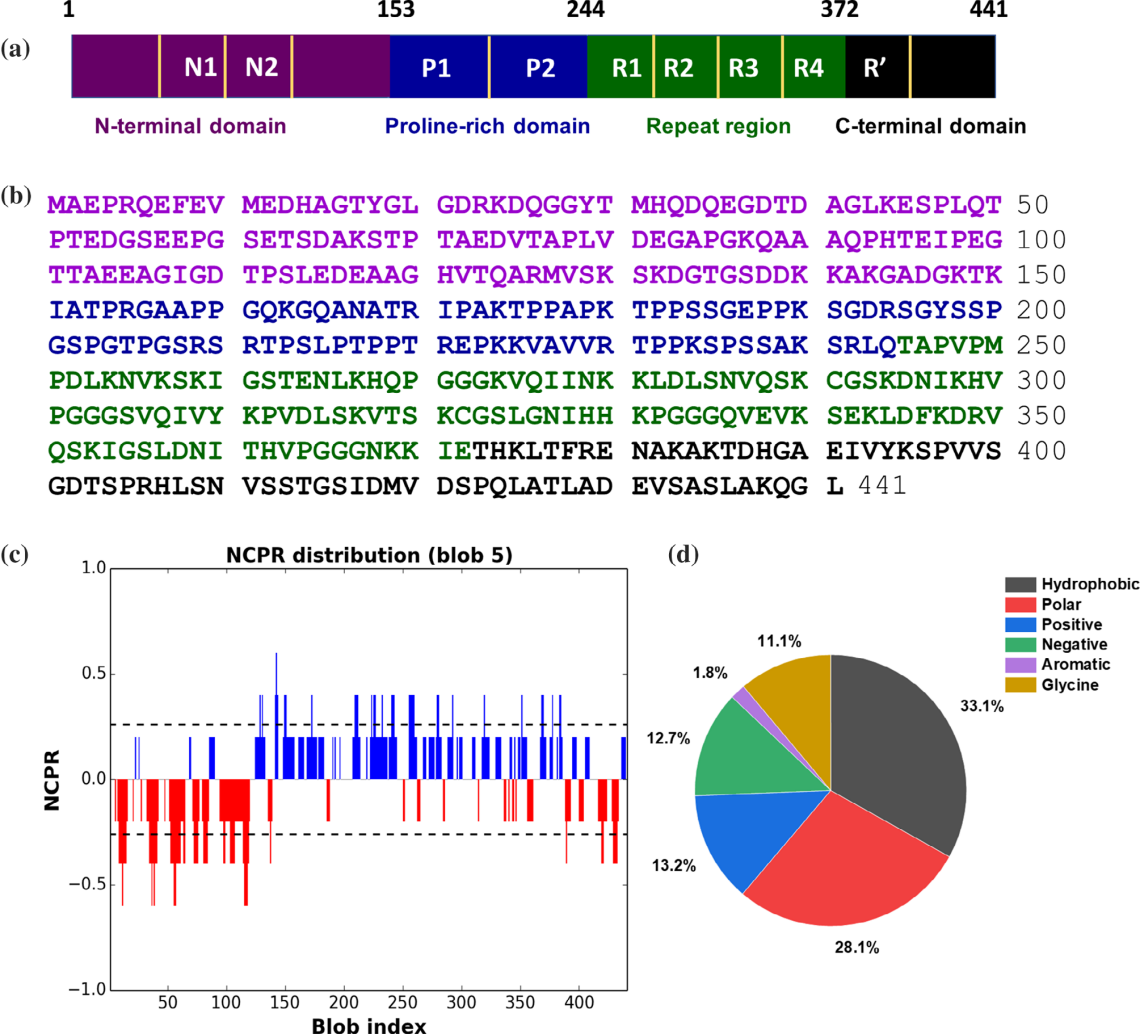
Amino acid sequence of tau. The sequence architecture (a) and the amino acid sequence (b) of full‐length tau (htau40). (c) Net charge per residue (NCPR) of tau (htau40), positive charges represented in blue whereas negative charges in red [generated using CIDER (Classification of Intrinsically Disordered Ensemble Regions) v1.7; Holehouse et al., 2015; Reference 119. (d) The amino acid composition of htau40 is shown as pie chart

Tau was first identified as a microtubule‐associated protein in the 1970s.^1^, ^2^, ^3^, ^4^ The protein received increasing attention after it was shown in 1986 that it is the major constituent of paired helical filaments (PHFs) and straight filaments (SFs) found in the brain of Alzheimer's disease (AD) patients.^5^, ^6^, ^7^, ^8^, ^9^, ^10^, ^11^, ^12^, ^13^ In addition, mutations in tau were identified in patients that were diagnosed with frontotemporal dementia, indicating that tau is a disease‐causing agent.^14^, ^15^, ^16^, ^17^, ^18^, ^19^ Today several neurodegenerative diseases have been identified, which result in insoluble tau deposits in diseased brains. These diseases have been classified under the name of tauopathies.^20^ The amino acid sequence of tau is encoded by the *MAPT* gene on chromosome 17.^21^ Alternative splicing of exons 2, 3, and 10 produces six isoforms of tau with different sequence compositions.^22^, ^23^ The nomenclature of these tau isoforms is based on the domains that are present in the amino acid sequence.^22^ The longest isoform, also known as full‐length tau, is a 45.9‐kDa intrinsically disordered protein (IDP) comprising two acidic insertions of 29 amino acids each (N1 and N2), a proline‐rich region, five pseudo‐repeats (from R1 to R4 and R'), and a C‐terminal tail (Figure 1). The full‐length human tau (htau40) is also denoted as 2N4R. The shortest isoform of tau, the embryonal htau23, comprises only three repeats and lacks the N‐terminal inserts. Isoforms containing all pseudo‐repeats are conventionally indicated as 4R, while those lacking the R2 domain are defined as 3R.^24^ Tau is found in different areas of the brain^10^ and the expression of the six isoforms is regulated according to the development of the nervous system^23^, ^25^: the expression of htau23 is up‐regulated in the fetal human brain,^22^ while at mature stages of development it is down‐regulated in favor of the 4R isoform.^20^

### 1.2 Physiological activities of tau

**FIGURE 2:**
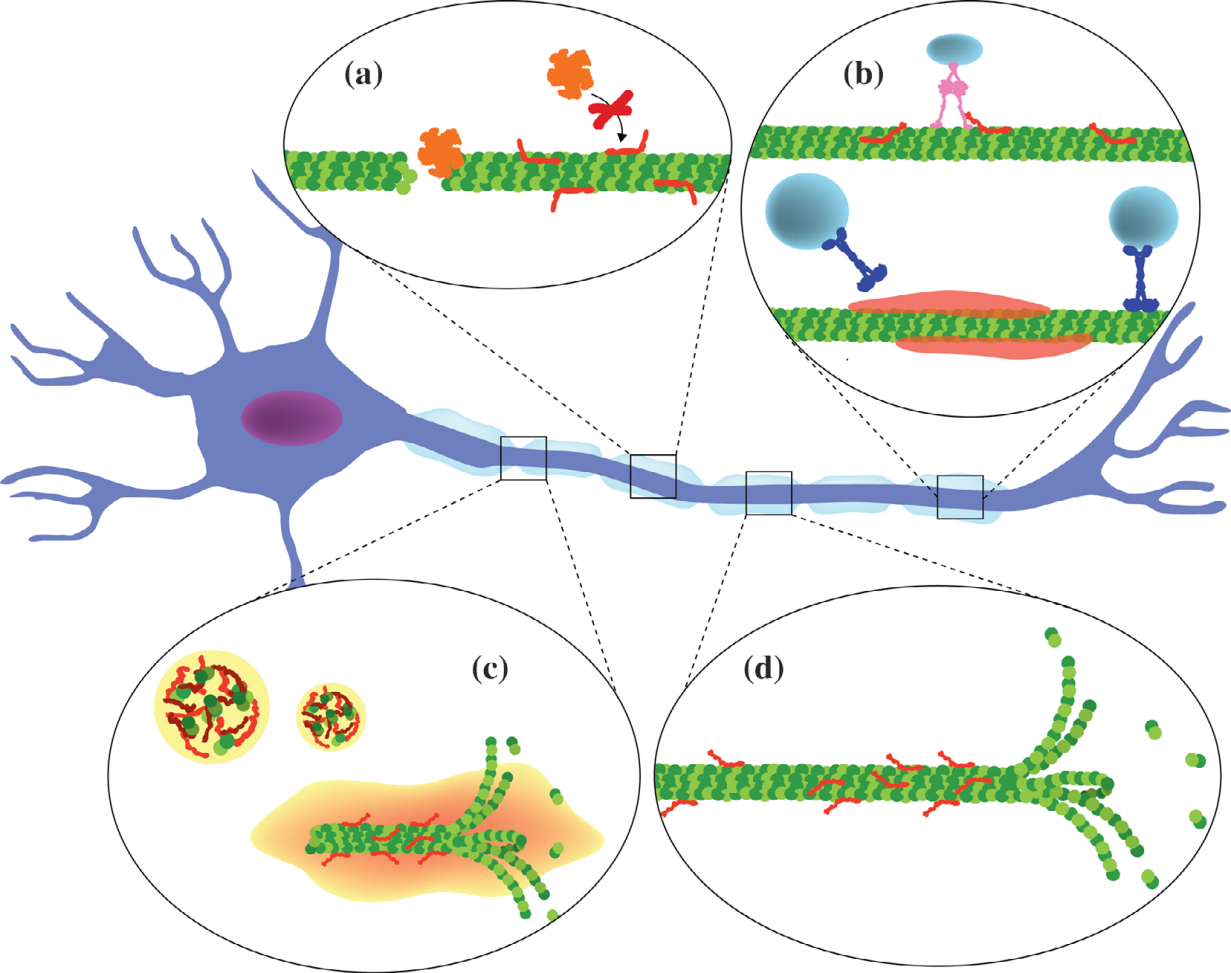
Cellular tau activities. (a) Tau (red) protects microtubules from the action of severing enzymes (e.g., Katanin, in orange). (b) Anterograde and retrograde axonal transport are regulated by the presence of tau on microtubules: tau can stabilize dynactin complexes (pink) on the microtubule surface, while the formation of tau condensates induces the dissociation of kinesin (blue). (c) The recruitment of tubulin into tau droplets promotes the growth of microtubules. (d) The binding of tau, mainly at their labile domains, stabilizes the filaments and protects them from catastrophe events, thus favoring their growth

The molecular factors and mutations that cause disease can broadly be grouped into two classes, those that lead to loss of physiological function and those that result in an aberrant activity termed gain‐of‐toxic‐function. In the context of tauopathies, it is therefore important to characterize and understand the physiological activities of tau (Figure 2). Indeed, tau detachment from microtubules and microtubule dysfunction have been connected to the dysfunction and death of neurons.^20^, ^26^

#### 1.2.1 Tau, microtubules, and phase separation

The binding of tau to microtubule filaments in axons was the first biological role attributed to tau.^1^, ^2^, ^3^, ^4^ In 1975, Weingarten et al. reported the isolation of a “protein factor” tau, which was found associated with microtubules, thus identifying the protein as belonging to the family of microtubule‐associated proteins.^1^ Since then, tau was shown to be involved in many aspects of microtubule formation and dynamics, including the promotion of tubulin polymerization,^27^, ^28^ elongation and stabilization of microtubule filaments,^2^, ^4^, ^29^ the spacing of microtubule filaments,^30^ as well as protection of microtubules from severing enzymes^31^, ^32^ (Figure 2). The interaction of tau with tubulin and microtubule filaments occurs via specific regions in the tau sequence: the proline‐rich region and the neighboring pseudo‐repeat domain in the C‐terminal half of the protein.^29^, ^33^, ^34^, ^35^, ^36^

**FIGURE 3:**
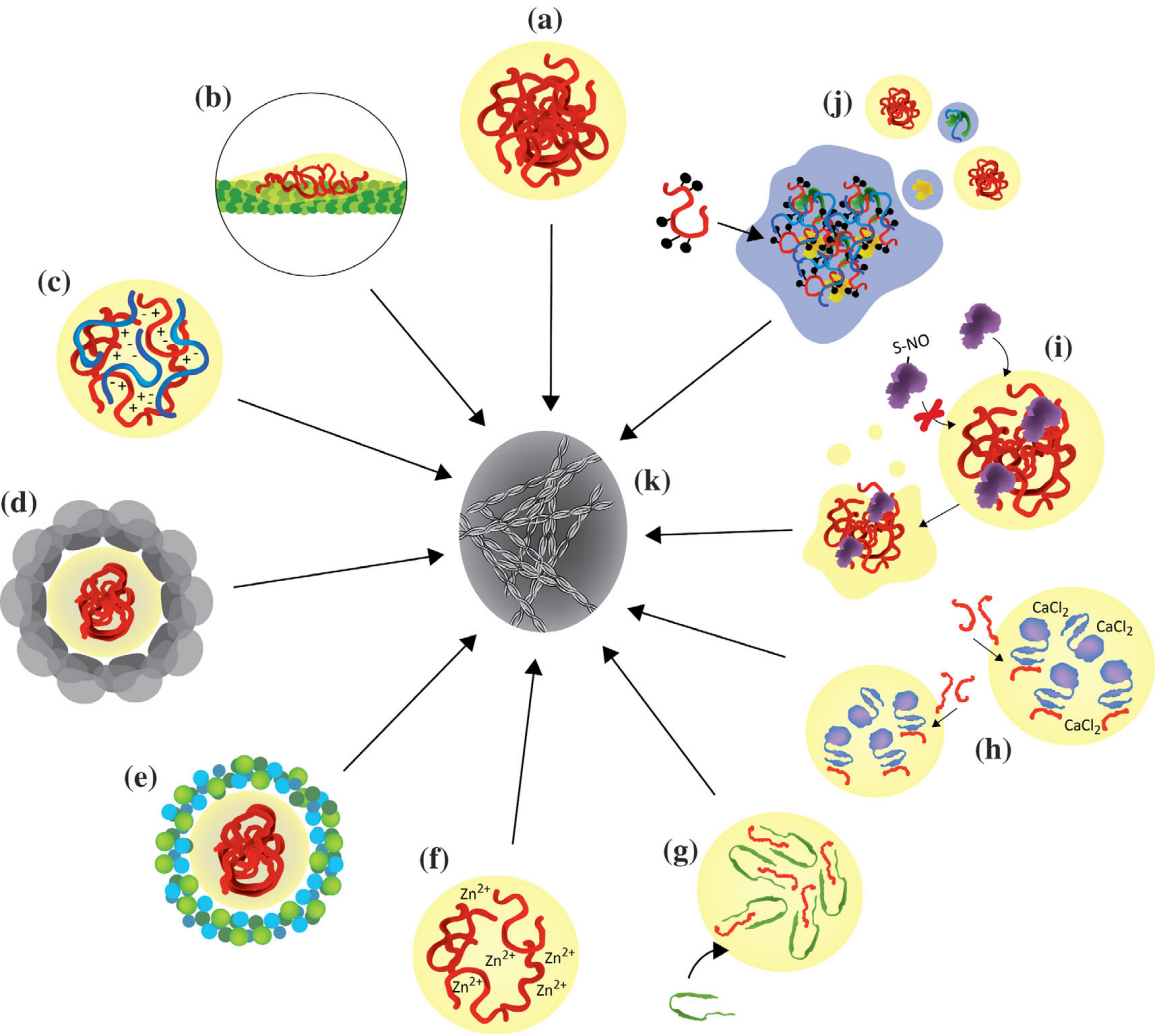
Tau liquid–liquid phase separation. (a) Coacervation of tau can occur in the absence of any factor, that is, simple coacervation. (b) When bound to the microtubule surface, tau can form condensates, also termed tau islands. (c),(d) The interaction with RNA molecules favors LLPS through electrostatic interactions (c), and as an effect of excluded volume, crowding agents, for example, PEG, Ficoll, Dextran, can enhance tau LLPS (d). (e),(f) LLPS of tau can also be induced by salting out, for example, at high NaCl concentrations (e) or in the presence of Zinc (f). (g) Alpha‐synuclein is recruited into tau droplets, which enhances its aggregation. (h) The calcium binding protein EFhd2 undergoes LLPS in the presence of CaCl_2_ and can recruit tau. When CaCl_2_ is missing, the droplets containing Efhd2 and tau shift towards fibrils. (i) The chaperone protein PDI1 regulates the dissolution of tau droplets upon its recruitment; the disease‐associated S‐nitrosylated form of this protein loses its function. Thus, droplets of tau do not dissolve and drift to pathological fibril formation. (j) Excessive tau LLPS can lead to the formation of SGs, and at the same time stress can induce hyperphosphorylation of tau, which is then recruited into SGs, where also RNA molecules and protein can interact with tau. (k) Despite the physiological functions of tau LLPS and droplet formation, aberrant maturation of the droplets can lead to fibrillization of tau

Tau promotes tubulin polymerization by decreasing the critical concentration for polymerization of tubulin into microtubules.^4^ The optimal temperature at which tubulin polymerizes is around 37°C.^37^ In the presence of tau condensates, microtubules also polymerize at lower temperatures (20–25°C), indicating that the protein acts as an enhancer of the nucleation process. More recently, Hernández‐Vega et al. showed that microtubule filaments can efficiently nucleate in vitro from droplets of phase‐separated tau, suggesting that tau condensates may act as independent tubulin nucleation centers^38^ (Figure 3). The ability of tubulin to polymerize from phase‐separated tau is inhibited by phosphorylation of tau at threonine 231, which is located in the proline‐rich region P2.^39^

Microtubules are characterized by “dynamic instability”,^40^ a mechanism through which microtubule filaments quickly assemble and/or disassemble, allowing fast cytoskeleton rearrangements according to the cellular needs.^41^ Tau, as other microtubule‐associated proteins, is considered to stabilize polymerized microtubules and to protect them from catastrophic events^42^ Tau binds to microtubules at the tubulin heterodimer interface.^36^ The binding of tau to microtubules decreases tubulin dissociation and the disassemble rate^29^ and provides stability to microtubules filaments.^42^ It was also observed that tau localizes to the labile part of microtubule filaments^43^ and promotes their polymerization by preventing the binding of stabilizers.^43^, ^44^

An emerging role is the tau‐mediated protection of microtubules from severing proteins.^31^, ^32^ Microtubule severing is an enzyme‐catalyzed mechanism, which causes internal breaks in the filaments and is important during mitosis, meiosis, neurogenesis, and other cellular events.^45^ Recent studies support the formation of tau condensates, termed tau islands that form on the surface of microtubules and protect them from the binding of severing enzymes, that is, katanin and spastin.^31^, ^46^, ^47^

#### 1.2.2 Neuronal development

Rearrangements of the cytoskeleton are necessary during neuronal development^48^ and microtubules are important for axonal growth.^49^ Through its ability to modulate microtubule polymerization and dynamics, tau can contribute to neuronal development.^25^ Consistent with the role of tau in neuronal development, tau isoforms are differently expressed during development.^23^ In vitro and in vivo studies further demonstrated the involvement of tau in the regulation of gene expression,^50^ spine growth,^51^ and neuron maturation.^52^ Some of these activities of tau, however, have been challenged.^53^, ^54^, ^55^

#### 1.2.3 Regulation of axonal transport

Tau influences the interaction of motor proteins with microtubules, thus modulating axonal transport.^56^, ^58^ The N‐terminal domain of tau binds to the dynactin complex and stabilizes its binding to microtubules.^56^ Formation of tau islands^47^ on the microtubule surface induces dissociation of kinesin‐1 motor proteins,^46^ while members of the kinesin‐8 motor protein family and the dynein‐dynactin complexes do not dissociate from microtubules but display pausing on their surface.^46^, ^47^ An influence on axonal transport might also be exerted by tau fibrils. LaPointe et al. suggested that in the filamentous state, the exposed N‐terminal region of tau interacts with the protein phosphates 1.^57^ The binding to tau might then contribute to the activation of protein phosphates 1, leading to dephosphorylation of the kinase GSK3‐beta. Once activated, GSK3‐beta phosphorylates kinesin, which inhibits its fast‐anterograde transport.^59^, ^60^

#### 1.2.4 Other tau activities

Several other activities have been attributed to tau and were summarized in a number of reviews.^61^, ^62^, ^63^ Tau might, for example, play a role in synapses where it interacts with the tyrosine kinase Fyn.^64^ The tyrosine kinase Fyn, which contains an SH3 as well as an SH2 domain, mediates tau phosphorylation via this interaction.^65^ Proteins containing SH3‐domains are also located on membranes^66^, ^67^ and tau was indeed found to interact with neuronal membranes.^68^ In addition, tau binds to filamentous actin^69^ as well as synaptic vesicles^70^ further supporting the role of tau in synapses and neuronal activity.^71^ Because of the intrinsically disordered nature of tau,^72^ many of these interactions are highly dynamic and can be regulated through post‐translational modifications.^73^, ^74^

### 1.3 Insoluble tau deposits

Tau aggregation is a hallmark in many neurodegenerative diseases, including AD, Pick's disease (PiD), frontotemporal dementia, and Parkinsonism linked to chromosome 17, Progressive Supranuclear Palsy, and Corticobasal Degeneration.^20^, ^75^ These diseases belong to the so‐called *“tauopathies”*, a term coined to underline the connection between cognitive impairment and the accumulation of aggregated tau in diverse areas of the brain.^76^, ^77^ Tau can aggregate into distinct inclusions, such as neurofibrillary tangles (NFTs), neuropil threads (NTs), Pick's bodies, and other forms of deposits.^20^ The reasons for the pathogenic aggregation of tau in neurons, as well as the basis for the morphological differences of the deposits, are not yet fully understood.

The brains of AD patients are characterized by the presence of NFTs and NTs, intracellular inclusions composed of amyloid‐like fibrils of misfolded, hyperphosphorylated forms of tau.^6^, ^11^, ^12^, ^22^ NFTs and NTs accumulate in the neuronal somata and the dendrites, respectively.^11^, ^78^ Presumably starting from the entorhinal cortex, tau fibrils spread to different brain areas following six stages as characterized by Braak and collaborators.^79^, ^80^ Recently, cryo‐electron microscopy (cryoEM) studies revealed the three‐dimensional structure of ex vivo fibrils of tau from AD patient brain.^81^ The cross‐β‐structure core of these fibrils is composed of the R3, R4, and part of R' pseudo‐repeats of tau.^81^ While both 4R and 3R tau isoforms can be found in AD, 3R isoforms are more predominant in PiD.^82^ In PiD, tau fibrils have two distinct conformations, wide and narrow filaments (previously described as straight filaments). According to the cryoEM structure, the cross‐β core of these fibrils is composed by the R1, R3, R4, and part of the R' pseudo‐repeat (Figure 1a).^83^ In Progressive Supranuclear Palsy, tau fibrils accumulate in round NFTs^84^ in neurons but are also present in oligodendrocytes and astrocytes.^20^

Supported by an increasing number of high‐resolution structures of ex vivo tau fibrils determined by cryoEM, the hypothesis that different tauopathies each correspond to a specific fibril conformation was proposed.^85^ The different tau fibril conformations are named strains. As in the case of prions, tau strains can spread from one cell to another and seed aggregation of monomeric tau.^86^, ^87^ As evinced by biochemical characterization of tau fibrils from human brains as well as studies in mice, tau strains have potentially a unique plethora of characteristics, including isoform composition of the fibrils, fibril conformation, distribution of tau pathology, and “aggressiveness” that is different propensity and efficiency to spread and to promote aggregation.^88^, ^89^, ^90^ Clinical and neuropathological hallmarks in tauopathies might thus be connected to the supramolecular structural properties of tau fibrils.

As to what is causing the acquisition of distinct fibril conformations, not only the isoform composition but also post‐translational modifications (PTMs) of tau, such as phosphorylation, ubiquitination, and acetylation, might have an impact on which structure tau adopts in amyloid fibrils.^91^, ^92^ The combination of cryo‐EM with mass spectrometry showed that tau fibrils from AD and Corticobasal Degeneration not only differ in their supramolecular structure but also in their PTM pattern, suggesting a possible link between PTM of tau and the formation of disease‐specific tau strains.^91^ PTMs have also been associated with different pathological behaviors of tau oligomers in AD. Phosphorylation on T231 and S235 was found to be correlated with higher seeding efficiency while singly phosphorylated oligomers displayed less ability to spread. The efficacy of oligomer‐based seeding was also correlated to clinical symptoms, suggesting that PTMs of soluble tau oligomers might influence the severeness of AD.^92^

## 2 PHYSICAL CHEMISTRY OF TAU LLPS

### 2.1 IDPs drive biomolecular condensation

Intrinsically disordered proteins/regions (IDPs/IDRs) have emerged as the major drivers of intracellular liquid–liquid phase separation (LLPS) into membrane‐less organelles.^93^, ^94^, ^95^ IDPs belong to the class of proteins that do not possess defined tertiary conformations and they adopt an ensemble of structures sampling a wide range of conformational space.^96^, ^97^ It is their structural plasticity that enables them to potentially engage in various biological functions such as cell signaling, transport, and so forth.^98^ Approximately one‐third of proteins comprising the eukaryotic proteome are either completely disordered or consist of disordered domains.^99^ While the folded globular proteins are known to follow the sequence‐structure–function paradigm, IDPs extend the tenets of this paradigm.^96^

With the recent flurry of emerging studies on membrane‐less organelles,^93^, ^100^, ^101^ it is becoming increasingly evident that IDPs/IDRs are the major constituents of these condensates because of their ability to form weak, transient, multivalent interactions.^102^, ^103^, ^104^ Efforts have been made to understand the effects of sequence‐specific features on the phase behavior of IDPs using both molecular simulations and experiments. The LLPS behavior of IDPs is primarily governed by charge–charge interactions,^95^, ^105^ π‐π and cation‐π interactions,^106^ hydrophobic contacts,^107^ charge patterning,^108^ and mingling of charged residues throughout the sequence.^109^ Often these sequences are described by a stickers‐and‐spacers architecture that controls phase transitions.^94^ Fused in sarcoma (FUS)^110^ and other FUS‐like RNA binding proteins such as TDP‐43,^110^ hnRNP A1,^111^ and hnRNP A2^112^ have been used to decipher the intriguing molecular language of LLPS. Additionally, aberrant phase transitions are associated with a range of neurodegenerative diseases.^113^

### 2.2 Sequence determinants of tau phase separation: Revisiting the tau sequence space

The intrinsic disorder and the phase separation propensity of tau are predicted by a range of bioinformatic tools including CatGranule,^114^ PONDR‐FIT,^115^ IUpred,^116^ PLAAC, and FuzPred.^117^ Its dynamic conformation is accounted by a high content of proline^118^ (9.7%, omega = 0.11446) and glycine (11%), polar and charged amino acids (FCR^119^ = 0.259; NCPR^119^ = 0.005) (Figure 1c,d). Tau undergoes LLPS in vitro under cell‐free conditions and in cells.^38^, ^120^, ^121^, ^122^, ^123^, ^124^ Recent studies have highlighted the critical roles of lysine residues^125^ (10%), the proline‐rich domain (PRD),^126^ the two oppositely charged termini,^127^ the aggregation‐prone hexapeptide region,^120^ and the KXGS^128^ motifs in the microtubule‐binding region (MTBR) for the LLPS of tau. The positively charged lysine residues can undergo post‐translational modifications that can regulate protein functions.^129^, ^130^ Lysine modifications can tune the overall charge of the polypeptide chain that can control the condensate formation from tau. Similar effects have been demonstrated for serine, threonine, and tyrosine residues that can undergo reversible phosphorylation.^39^, ^123^ Since the majority of phosphorylation sites are located in the proline‐rich domain, the LLPS propensity is modulated by this domain.^126^ Additionally, aggregation‐prone hexapeptides (^275^VQIINK^280^ and ^306^VQIVYK^311^), can serve as local nucleation sites during phase separation of the repeat domain (tau K18).^120^, ^128^ Previous studies have demonstrated that isoforms of tau show different phase separation propensity. This difference in phase separation ability can be correlated with the charge on these isoforms, and hence the change in the pI, presence or absence of inserts in the N‐terminal part, and the number of repeat regions. Full‐length tau (htau40, pI 8.42) has a higher phase separation propensity than any other isoforms under near‐physiological conditions, as evident from the lower critical threshold concentration and time required to undergo phase separation, compared to K18 (4R) and K19 (3R).^120^, ^125^

### 2.3 Intermolecular interactions modulating phase separation: Electrostatic and hydrophobic interactions

LLPS is a physiochemical process that is driven by multivalent weak interactions resulting in a protein‐rich dense phase and a protein‐depleted light phase.^131^ The favorable interactions within and between proteins and with nucleic acids that overcome the entropic penalty to LLPS include electrostatic, cation‐π/π‐π, hydrophobic interactions, and so forth.^104^, ^132^, ^133^ Hydrophobic interactions are contributed by aromatic (F, W, Y), non‐polar (A, I, V, L, M, G, and P), and sometimes charged amino acids (context‐dependent). These amino acids can cause the polypeptide backbone to exhibit favorable chain‐chain interactions over chain‐solvent interactions.^105^ Additionally, crowding agents such as polyethylenglycol (PEG) and Ficoll (volume exclusion effect) or salt (salting out/Hofmeister effect) can promote LLPS.

The tau sequence contains a small number of aromatic amino acids. Besides, tau is an amphipathic protein in which the charged residues are distributed non‐uniformly and cluster in different domains: a negatively charged N‐terminal region (1–150 aa), the strongly positively charged proline‐rich region (151–243 aa), a mildly positive repeat domain (244–372 aa), and a slightly negative C‐terminal region (373–441 aa).^3^, ^4^, ^24^, ^134^ In recent studies of tau LLPS, experiments were performed at physiological and semi‐physiological conditions, comprising unmodified, post‐translationally modified, and N‐terminally and C‐terminally truncated parts of tau, with cofactors (RNA, heparin, metal ions) forming two different types of condensates termed as simple coacervates and complex coacervates, respectively.^39^, ^120^, ^121^, ^122^, ^123^, ^124^, ^125^, ^127^, ^128^ Both simple and complex condensates have been found to be sensitive to increasing ionic strength, where the addition of 100 mM NaCl can dissolve the tau droplets. This finding suggested that the phase separation of full‐length tau is driven by electrostatic interactions between the N‐terminal projection domain and the mid‐region, anchored by the proline‐rich region of tau (Figure 1). The importance of electrostatic interactions for tau LLPS is further supported by the observation that LLPS occurs spontaneously when the N‐terminal truncated part (tauΔ1‐117) is mixed with the negatively charged tauΔ118‐441 truncation. Due to the net negative charge, the tau N‐terminal region is likely to provide electrostatic contributions to LLPS as evinced by studies on the full‐length protein and its variants.^38^, ^39^, ^135^ Turbidity measurements combined with fluorescence microscopy showed that the tau construct, K25, comprising only the N‐terminal projection domain and proline‐rich domain, forms liquid‐like droplets.^123^, ^125^ By testing the droplet formation of N‐terminal mutated tau at different NaCl concentrations, the LLPS propensity decreased at higher ionic strength. Consistent with these observations, the removal of the N‐terminal domain abolished the LLPS ability.^123^ Thus, the N‐terminal region is likely to play an important role in tau LLPS by establishing electrostatic interactions with the positively charged central part of tau. Tau contains some hydrophobic amino acids mainly clustered in the K18 region (Figure 1). While the low salt and truncation experiments emphasized the role of electrostatic interaction in tau LLPS, the fact that K18 droplets can be dispersed by the addition of 1,6‐hexanediol hinted at the role of weak hydrophobic interactions.^120^, ^128^ Hydrophobic interactions become more evident in the case of high‐salt tau droplets in which electrostatic repulsions are screened by salt, or in the post‐translationally modified tau in which the net positive charge of tau is reduced by the addition of negative groups upon phosphorylation.^123^, ^140^ As described above, lysine residues play a role in promoting tau LLPS.^125^ Less attention, on the other hand, has so far been paid to the contribution of arginine residues in tau LLPS. When studying the contribution of lysine and arginine to RNA‐induced phase separation, Ukmar‐Godec et al. reported that the side chains of both amino acids can establish hydrogen bonds with RNA.^125^ While the guanidinium group in arginine provides stronger interactions within RNA condensates, the RNA‐interactions of lysine side chains grant more flexibility due to the lower strength of the corresponding hydrogen bonds.^125^ Therefore, an intriguing interplay of electrostatic, hydrogen bonding, and hydrophobic interactions drive homotypic and heterotypic LLPS of tau.

### 2.4 Thermoresponsive phase behavior of tau ‐ LCST and UCST transitions

**FIGURE 4:**
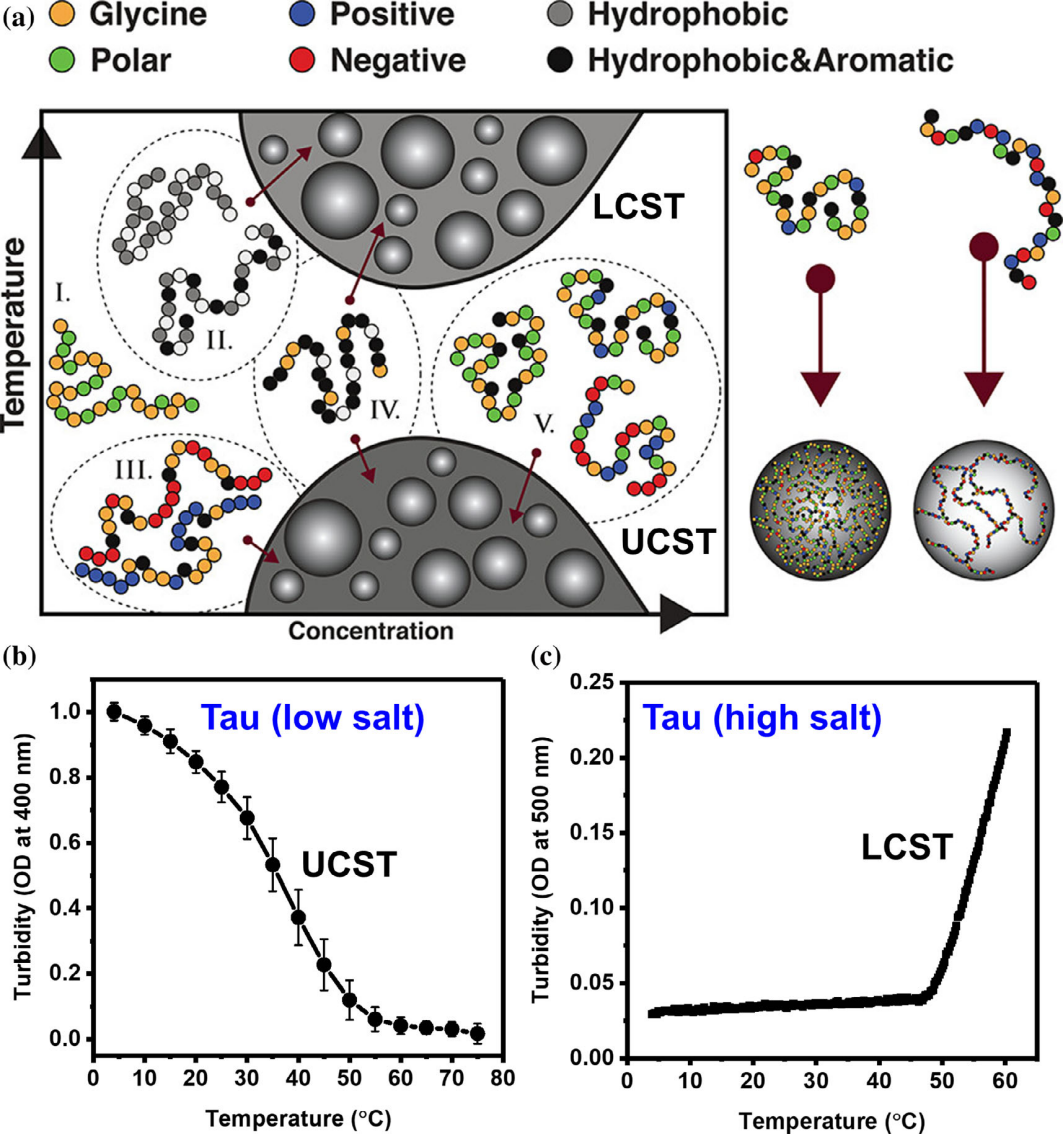
Thermo‐responsive phase behavior. (a) IDPs enriched with polar and charged amino acids demonstrate UCST transitions, whereas, the chains depleted of these amino acids and supplemented with hydrophobic residues undergo LCST transitions. Adapted from Reference 132 Martin, E.W. et al., (2018) with permission from ACS. (b), (c) Temperature‐dependent phase behavior of tau. The low‐salt droplets of full‐length tau exhibit a UCST transition (b). Full‐length tau at high salt (3.5–4.5 M) undergoes an LCST transition (c). Adapted from Reference 139 Boyko, S et al. (2019) and Reference 140 Lin, Y et al. (2020) with permission from Elsevier

IDPs such as tau can experience context‐dependent, two types of thermoresponsive phase transitions. These transitions are primarily driven by the network of weak multivalent interactions between short motifs via transient or reversible physical contacts.^132^, ^136^, ^137^ One phase to two‐phase transitions occurring above a fixed temperature, known as critical temperature (θ_lc_), are referred to as lower critical solution transitions (LCST), whereas, transitions taking place below a particular temperature (θ_uc_) are characterized as upper critical solution transitions (UCST). Previous studies have established a connection between the thermoresponsive behavior and the amino acid sequences of polypeptides (Figure 4a). In a nutshell, the high content of polar residues together with aromatic residues favors UCST behavior, while polypeptides depleted in charged amino acids and enriched with hydrophobic residues are ideal for LCST transitions.^95^, ^132^, ^138^ However, the thermoresponsive phase behavior of a polypeptide also depends on the sequence distribution of oppositely charged patches, because they can modulate chain‐solvent interactions. The driving force for UCST transitions is molecular interactions (ΔH) that dominate with decreasing temperature over the entropic term (TΔS).^105^, ^136^ These interactions involve electrostatic interactions, cation‐π interactions, and π‐stacking. On the other hand, LCST transitions are primarily driven by hydrophobic interactions between the bulky apolar side chains of amino acids resulting in the expulsion of solvating water molecules from the vicinity of the polypeptide making this transition favorable. The rise in the temperature favors this LCST transition due to an increase in the entropic term (TΔS).

Tau protein being ampholytic has been shown to undergo intriguing context‐dependent LCST or UCST transitions.^120^, ^139^, ^140^, ^141^ Tau coacervates in low salt conditions demonstrate UCST behavior due to enthalpically driven interactions (Figure 4b,c). In contrast, high salt coacervates of full‐length as well as K18 variants of tau exhibit LCST transition due to the involvement of entropic contributions. At high ionic strength, the charges on the protein are screened, and therefore, the intermolecular interactions are primarily governed by entropically driven hydrophobic interactions. Similarly, though electrostatic interaction promotes complex coacervates of tau, it is the gain in the solvation entropy that regulates phase transition as evident from the LCST behavior of tau‐RNA and tau‐heparin coacervates. Taken together, the thermoresponsive phase behavior of tau can be modulated by various factors such as pH, ionic strength, protein concentrations, chain length, mutations, and post‐translational modifications.

### 2.5 Conformational dynamics of tau within condensates

**FIGURE 5:**
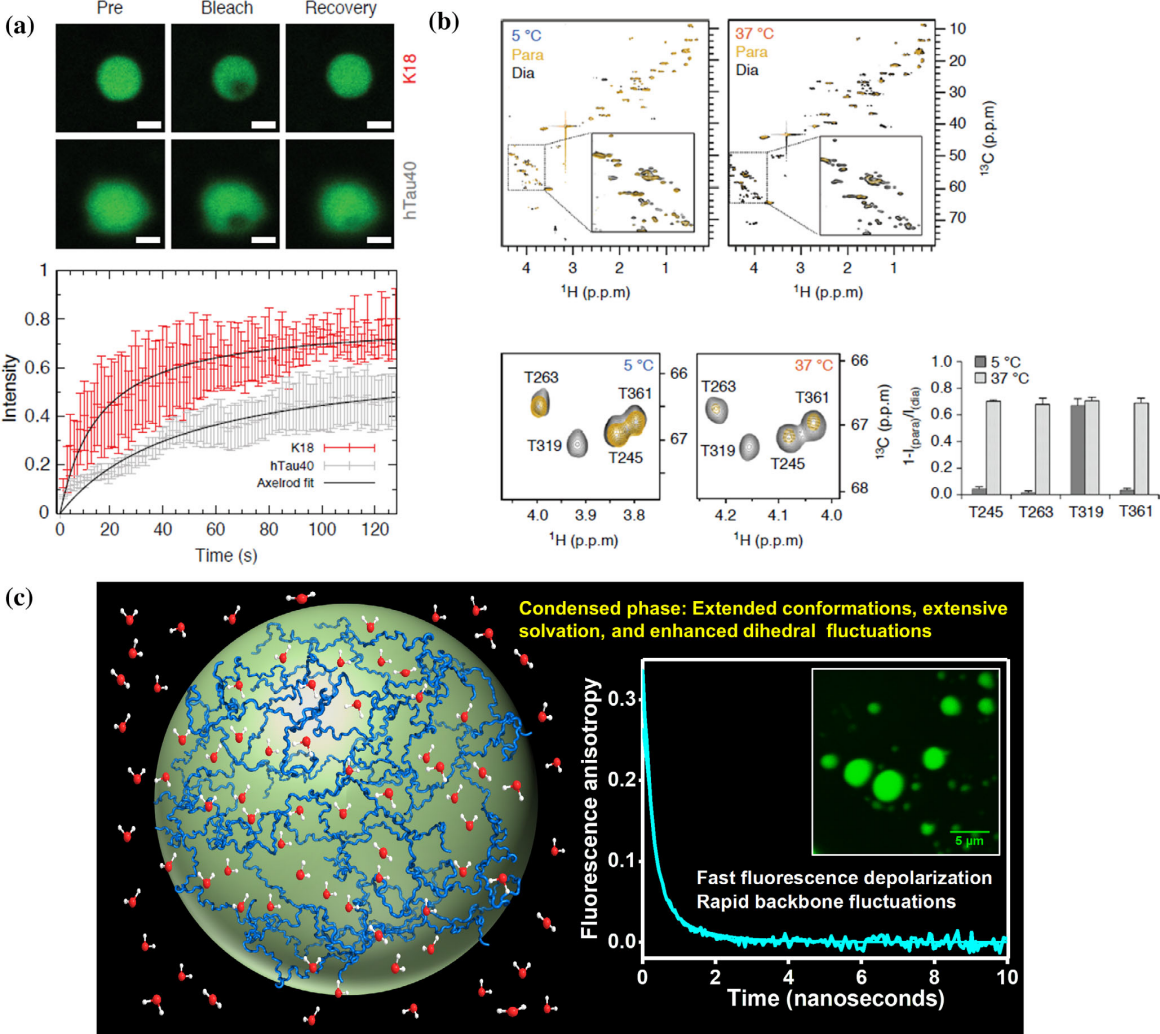
Conformational and dynamic properties of tau in phase‐separated condensates. (a) FRAP images and normalized FRAP recovery curves for hTau40 (gray) and K18 (red) droplets in the presence of RNA. Adapted from Reference 125 Ukmar‐Godec, T et al. (2019). (b) Paramagnetic broadening in K18 (tagged with MTSL at native cysteines), quantified in 2D ^1^H‐^13^C HSQC spectra at 5°C (left) and 37°C (right). Paramagnetic and diamagnetic states are represented by gold and black, color respectively. Insets show the highlighted Cα‐Hα region of each spectrum. Selected region of the ^1^H‐^13^C HSQC spectra shown in upper panel, highlighting paramagnetic broadening of the four threonine residues in K18 (lower panels). The histogram displays the quantification of paramagnetic broadening observed in ^1^H‐^13^C HSQC spectra at 5°C and 37°C. Adapted from Reference 120 Ambadipudi, S et al. (2017). (c) Conformational expansion, fluctuation, and solvation during phase separation of tau K18. Adapted from Reference 124 Majumdar, A et al. (2019) with permission from ACS

The highly dynamic liquid‐like nature of biomolecular condensates is thought to be critical for achieving cellular functions. The dynamical shift can cause aberrant phase transitions resulting in pathological protein aggregation. There is therefore a great need to better understand the conformational dynamics within biomolecular condensates. Various modes of protein dynamics over a wide range of timescales from picoseconds to seconds can be determined using a diverse array of time‐resolved readouts both in spectroscopic and microscopic formats (Figure 5). For instance, fluorescence recovery after photo‐bleaching (FRAP) kinetics and fluorescence correlation spectroscopy can be used to study the translational diffusion of proteins within the condensates. In addition, fluorescence anisotropy measurements can offer important insights into the rotational dynamics and backbone dihedral mobility of IDPs on the picosecond to nanosecond timescales.^97^ These studies suggested that high intrinsic disorder, flexibility, and mobility are present in the condensed phase of tau (Figure 5).^120^, ^121^, ^128^, ^142^ By following the excimer emission of pyrene, an intramolecular proximity probe, Majumdar et al. showed a structural expansion of the tau construct K18 upon LLPS.^124^ Such an expansion might favor the multivalency that promotes weak but dynamic interchain interactions. The phase separation of tau K18 was also associated with extensive solvation and fast dihedral fluctuations as evident by enhanced fluorescence quenching and fast fluorescence depolarization, respectively (Figure 5c).

Electron paramagnetic resonance (EPR) was also used to gain insight into the conformational properties of tau in liquid‐like droplets. Constant‐wave EPR experiments on the tau construct Δtau187 showed similar line‐shapes in both solution and the droplet state.^121^, ^142^ This line‐shape also differed from the one obtained when heparin was added to the tau solution, that is, when aggregation was induced.^121^ Double electron–electron resonance (DEER) experiments on frozen samples were furthermore used to probe the mean distance flanking the hexapeptide in the R3 repeat: while in conditions of aggregation this region adopts an open conformation,^143^ this was not the case in conditions of droplet formation.^121^

Nuclear magnetic resonance (NMR) spectroscopy can provide residue‐specific or even atomic resolution structural insights into the conformational dynamics of biomolecules under physiological conditions. It is therefore optimally suited to dissect the conformational and dynamic properties of proteins and nucleic acids in the condensate state.^144^, ^145^ In addition, NMR spectroscopy has been critical for the determination of conformational ensembles of IDPs such as tau.^72^, ^146^, ^147^, ^148^, ^149^ Using paramagnetic probes in NMR experiments, it was shown that the repeats of tau come closer in space, an interaction which could be established within one tau molecule (intramolecular interactions) or between repeat domains of more tau molecules (intermolecular)^120^ (Figure 5). The analysis of carbon detected NMR experiments further indicated that the residues in the KXGS motifs, which are located in the repeat domain, adopt a β‐hairpin‐like state in K18 droplets.^128^ During droplet maturation, such folding events can potentially drive the aggregation of tau.

## 3 TAU CONDENSATION: FROM COFACTORS TO POST‐TRANSLATIONAL MODIFICATIONS

### 3.1 Complex coacervates of tau: RNA, heparin, and ligands

IDPs can undergo LLPS through self‐coacervation driven by intra‐ and intermolecular interactions^95^, ^150^, ^151^, ^152^ and can be assisted in their condensation process by other molecular factors such as crowding agents, polymers, and ions.^153^, ^154^ Also, IDPs are prone to charge‐driven complex coacervation with nucleic acids.^155^ While both DNA and RNA can promote IDP condensation, many studies have focused on RNA molecules which play key roles in the assembly of membrane‐less compartments through their binding to proteins containing RNA‐binding domains.^152^, ^153^ As a direct consequence of its negative charge, RNA can induce and regulate the formation of condensates by complex coacervation through compartmentalization of proteins or by functioning as a scaffold platform for protein–protein interactions.^155^

Tau interacts with a wide range of RNA molecules in vitro and in cells.^121^, ^125^, ^142^, ^156^ The affinity of tau for RNA was estimated as ~500 nM.^121^ RNA efficiently induces and promotes tau LLPS in vitro^120^, ^121^, ^135^ (Figure 3). Because tau and RNA molecules are positively and negatively charged, respectively, the tau/RNA interaction has a strong electrostatic component. In agreement with the importance of charge–charge interactions, tau LLPS in the presence of RNA is insensitive to 1,6‐hexanediol, an aliphatic alcohol that can dissolve droplets held together by hydrophobic interactions.^157^ Additionally, conditions in which charge neutralization occurs promote tau/RNA LLPS.^121^, ^135^, ^142^ Several different tau constructs/fragments can therefore undergo RNA‐induced LLPS when the tau/RNA molar ratio is optimized.^121^, ^135^, ^142^

Polyanions such as RNA and heparin have been widely used to overcome the electrostatic repulsion between tau molecules and induce tau fibrillization.^158^ In agreement with a close connection between LLPS and protein aggregation,^151^, ^159^, ^160^, ^161^ the addition of heparin to self‐coacervated K18 droplets resulted in fibril‐like structures.^120^ Moreover, physicochemical conditions that favor tau LLPS (low ionic strength, physiological pH, and temperature) also promote the heparin‐induced tau fibrillization, suggesting that similar interactions are important for both processes.^120^ Independent experiments further showed that the addition of heparin to phosphorylated full‐length tau not only induced LLPS, but upon longer incubation also results in the formation of fibrils.^123^

As is the case in many other systems, both tau self‐coacervation and RNA‐induced LLPS are promoted in the presence of molecular crowding agents such as dextran, PEG, and Ficoll^38^, ^39^, ^47^, ^120^, ^123^, ^125^, ^140^ (Figure 3). While the molecular mechanism underlying the influence of molecular crowding agents on LLPS has not yet been fully resolved, excluded volume and solvation effects are believed to contribute to the promotion of LLPS by molecular crowding agents.^154^ Notably, molecular crowding agents are not only useful to decrease the protein concentrations required for in vitro LLPS, but also allow mimicking the highly crowded environment inside cells.

Tau LLPS can be further modulated by metal ions and salts^140^, ^162^ (Figure 3). For example, zinc (Zn^2+^) lowers the critical concentration of tau for LLPS.^162^ In addition, tau condensation (in the absence of RNA/DNA) is favored at high salt concentrations that is by the addition of salts belonging to the Hofmeister series.^140^ Salting out by Hofmeister salts is known to decrease the solubility of proteins and potentially favor hydrophobic interactions. In contrast to Tau‐RNA complex coacervates, droplets derived from high salt‐induced tau phase separation were sensitive to 1,6‐hexanediol and undergo irreversible maturation causing tau fibrillization.^140^

### 3.2 Tau LLPS and protein interactions

Membrane‐less organelles such as stress granules consist of a large number of proteins and RNAs.^155^ Both RNA and other proteins can accordingly modulate protein LLPS.^152^ For example, the calcium‐binding protein EFhd2 interacts with tau through its coiled‐coil domain^163^ and has been found associated with tau aggregates in mouse models.^163^ When EFhd2 undergoes phase separation in presence of PEG and CaCl_2_, tau can colocalize within these droplets; but in the absence of CaCl_2_, EFhd2 influences tau droplets to shift towards solid‐like structures.^164^ Similarly, the chaperone protein disulfide isomerase (PDI) is recruited into tau droplets and promotes their dissolution.^165^ Notably, the S‐nitrosylated form of this chaperone protein is linked to neurodegenerative conditions and present in NFTs.^166^ Once S‐nitrosylated, PDI cannot be recruited into tau droplets and thus cannot regulate their dissolution.

Recruitment of one protein to condensates of another protein might also provide the basis for synergistic aggregation of the two proteins. For example, in Parkinson's disease with dementia, patients have not only insoluble deposits of α‐synuclein, but up to 50% of patients also develop tau‐containing neurofibrillary tangles.^167^ While α‐synuclein itself has a low propensity for self‐coacervation in physiological conditions, it readily concentrates in a phosphorylation‐dependent manner in tau droplets^168^ (Figure 3). In addition, α‐synuclein fibrils associate with tau droplets.^168^ Because high concentrations of both tau and α‐synuclein are reached in the droplets, their aggregation is promoted.

### 3.3 Post‐translational modifications regulating tau phase separation

Post‐translational modifications (PTMs) play a pivotal role in regulating LLPS by changing the protein's net charge, conformation, or ability to interact with other partners.^169^ Being intrinsically disordered, tau is highly susceptible to post‐translational modifications including phosphorylation, acetylation, glycosylation, glycation, and ubiquitination.^74^ PTMs influence the physiological^59^, ^170^, ^171^ and pathological^172^, ^173^, ^174^, ^175^, ^176^ activities of tau and also change the protein's propensity to undergo phase separation.^39^, ^120^, ^122^, ^123^, ^125^

Tau phosphorylation can occur on serine, threonine, and tyrosine residues, particularly represented in the tau sequence: 45 serines, 35 threonines, and 5 tyrosines are present in htau40, the longest isoform of tau.^1^, ^2^, ^22^ These residues can be phosphorylated by several kinases.^177^, ^178^, ^179^ In particular, proline‐directed protein kinases phosphorylate serine and threonine residues in the proline‐rich region,^24^, ^178^ while non‐proline‐directed kinases are responsible for the phosphorylation of serine and tyrosine in the pseudo‐repeat region and at the C‐terminus.^39^, ^178^, ^179^

Phosphorylation favors tau phase separation by addition of negative charges and decreasing the overall positive charge of tau.^39^, ^123^, ^169^ This lowers the isoelectric point (pI) of the protein and hence favors compactness in the chain such that the side chains of hydrophobic amino acids can fall under the van der Waals radii and establish interactions. High LLPS propensity was observed for strongly phosphorylated full‐length tau produced in SF9 insect cells,^123^ for tau and K18 phosphorylated in vitro by MARK2^120^, ^123^ as well as for AT180‐ and AT8‐phosphorylated full‐length tau.^39^, ^180^ Phosphorylation by the tyrosine kinase c‐Abl did not promote LLPS, but also did not decrease tau's ability to undergo phase separation.^39^ Since the aforementioned kinases target different residues spread in the tau sequence, these observations suggest that both the overall degree of phosphorylation and the specific site/sites of modification are important factors in PTM‐modulated tau LLPS. Site‐specific changes modulating tau LLPS might include PTM‐mediated changes in tau conformation.^149^ PTM‐mediated structural changes may also be responsible for the maturation of droplets into fibrils, thus offering one possible explanation for the shift from physiological to pathological aspects of tau LLPS.^180^

Another important disease‐associated post‐translational modification of tau is the acetylation of lysine residues.^181^, ^182^ A large number of lysine residues of tau can be efficiently acetylated *in vitro* by the acetyltransferases p300 and CREB.^183^ The addition of an acetyl group to the amide of lysine residues neutralizes the positive charges of the side chain and increases their hydrophobicity. Acetylation can thus attenuate or block LLPS, through disruption of electrostatic interactions mediated by lysine residues.^122^, ^184^ Indeed acetylation of tau strongly attenuated the ability of htau40 to phase separate and dissolved preformed htau40/RNA droplets.^122^, ^125^ Besides changes in the overall charge of tau, this is probably also caused by the disruption of hydrogen bonds between lysine side chains and RNA. Acetylation also interfered with RNA‐induced LLPS of K18.^125^ Thus, while both phosphorylation and acetylation decrease the positive charge of tau, they can have distinct consequences on tau LLPS. The effect that these two PTMs have on tau LLPS can also translate into their effect on the pathological aggregation of tau. For example, acetylation of lysine residues attenuates tau binding to RNA and thereby RNA‐associated complex coacervation of tau, but at the same time might favor the stacking of β‐parallel strands in tau amyloid fibrils.^91^ Hyperphosphorylation of tau has been shown to drive the maturation of tau droplets into a gel‐like state.^123^ Thus, PTMs might play a pivotal role in shifting the equilibrium from LLPS to aggregation of tau into insoluble deposits.

## 4 PHASE TRANSITIONS OF TAU IN PHYSIOLOGY AND DISEASE

### 4.1 Tau LLPS and microtubules

Recent findings correlated tau LLPS to its physiological interaction with tubulin and its ability to promote microtubule polymerization.^38^, ^39^, ^46^, ^47^ Hernández‐Vega reported that upon tau phase separation, and in presence of 1 mM GTP and 10 μM of tubulin, microtubules nucleate out from the droplets.^38^ Following this observation, Savastano et al. showed that this ability is inhibited by disease‐associated phosphorylation of T231 in the proline‐rich region. Phosphorylation at this site can induce the establishment of salt bridges between the phosphorylated T231 and its preceding R230, thus inhibiting microtubule‐promoting interactions with tubulin.^39^ Additionally, acetylation of tau attenuated its interaction with tubulin and its ability to promote the growth of microtubule filaments from the inside of tau droplets.^122^

Earlier, tau interaction with microtubules was thought to be able to induce tau aggregation as a consequence of the protein's overloading on the surface of microtubules.^185^, ^186^, ^187^ Indeed, microtubules may be considered as platforms that favor phase transitions in cells.^188^ Consistent with this hypothesis, fluorescence microscopy showed that tau can form condensate‐like islands on the microtubule surface.^46^, ^47^ The microtubule‐associated tau condensates share some of the liquid‐like properties observed for tau droplets in solution. Notably, the tau islands form at very low tau concentrations and were found to be involved in the interaction with motor proteins located on the surface of the microtubule.^46^, ^47^ Tau condensates can also recruit proteins involved in the establishment of microtubules architecture, for example, the plus‐end tracking protein EB1.^126^

### 4.2 Stress granule association of tau

**FIGURE 6:**
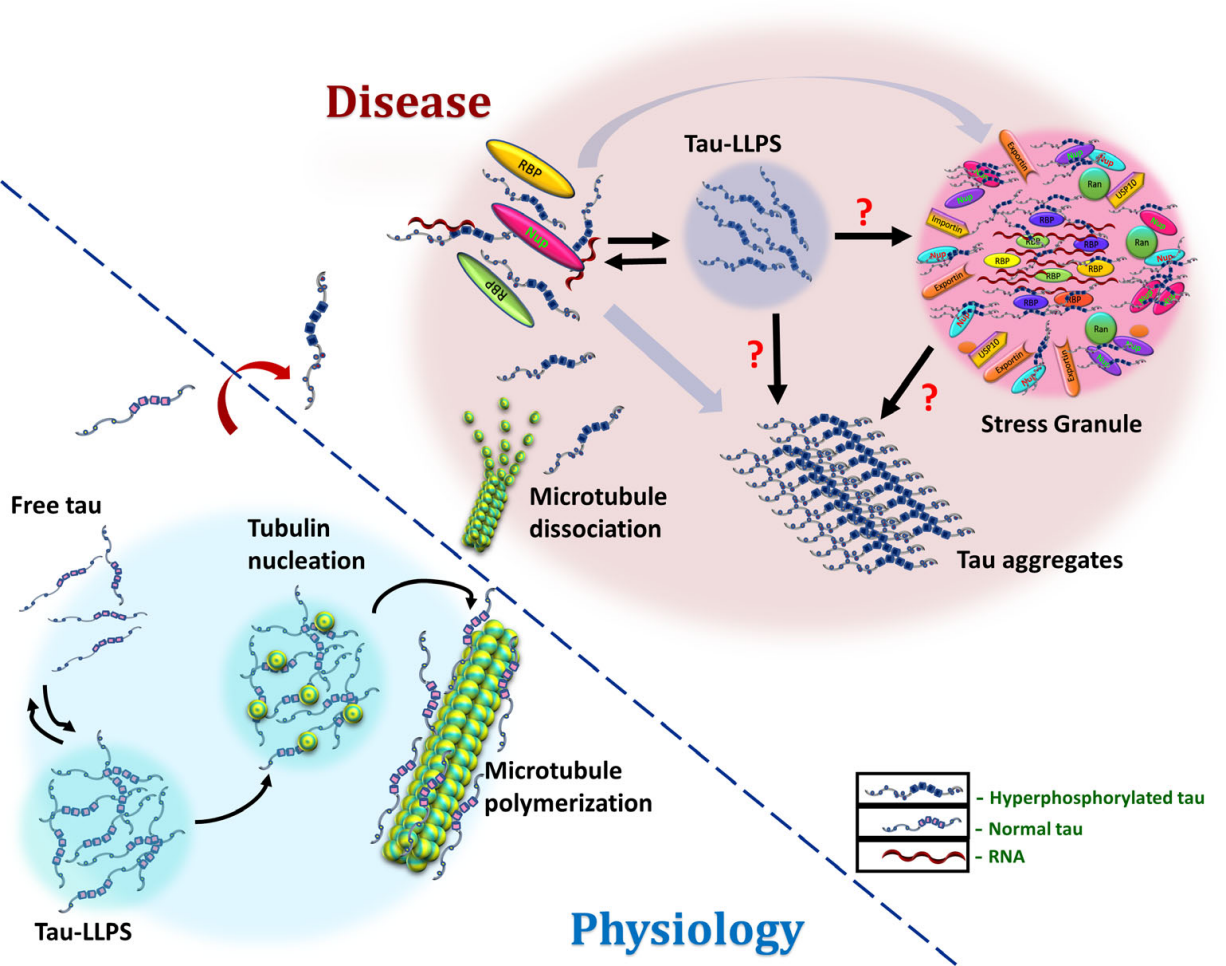
Phase transitions of tau in physiology and disease. Under physiological conditions, tau nucleates microtubule polymerization and stabilizes microtubule structure.^87^ Nucleocytoplasmic transport is active and normal. In pathological conditions, various etiological factors lead to hyperphosphorylation of tau (88) that eventually initiate its dissociation from microtubules.^89^ Furthermore, impairment of nucleocytoplasmic transport might lead to progressive accumulation of RNA, RBPs including TIA‐1, and nucleoporins such as Nup98 in the soma where interactions with mislocalized hyperphosphorylated tau potentially drive its phase separation.^205^ Tau condensates could then act as templates for the formation of either pathological stress granules or toxic fibrils. Stress granules on the other hand might also mediate the process of liquid–liquid to the liquid–solid transition of tau into pathogenic aggregates

Stress granules (SGs) are dense membrane‐less sub‐compartments in the cytosol, that comprise translationally silent mRNAs, certain translation factors, and several RNA‐binding proteins (RBPs) including FUS, T‐cell intracellular antigen 1 (TIA‐1), and poly(A)‐binding protein (PABP) that are known to aggregate via glycine‐rich domains.^13^ SGs assemble transiently in response to several stress conditions to aid the survival of the cell. Stress granule formation appears to be regulated by tau. Contrarywise, pathological changes related to tau are stimulated by the formation of stress granule. Aggregation of tau has been hypothesized to have proceeded through the pathway of stress granule formation^13^ (Figure 6). Stress leads to hyperphosphorylation of tau followed by its mislocalization to the soma and the dendrites where it interacts with stress granule‐associated RNA along with RBPs.^189^, ^190^

A close link of several SG‐associated RBPs with neurodegenerative diseases is known with a significant association of tau with the core‐nucleating protein TIA‐1. Nucleation by TIA‐1 promotes the assembly of secondary RBPs to generate a matured SG, which is a vital element of translational suppression under stress. The binding of TIA‐1 with tau is reported to contribute to its aggregation leading to disease‐related tau‐driven toxicity and neurodegeneration.^191^, ^192^, ^193^, ^194^ Tau similarly drives SGs aggregation as do RBPs during prolonged or repetitive phase separation events.^150^, ^153^, ^192^, ^195^ The analogous biophysical characteristics of tau and RBPs, the potency of tau to drive SG formation, and its propensity to aggregate in the vicinity of SGs, provide evidence for a strong association of tau with SGs. Tau is reported to co‐localize with SG‐associated RBPs,^196^ which might lead to the maturation into obstinate pathological SGs during stress. The association of ubiquitin with neurofibrillary tangles is also well documented,^197^ in line with shreds of evidence related to Ubiquitin‐specific protease 10 (USP10) as a crucial factor for the formation of SGs comprising tau, TIA‐1, and USP10.^198^ USP10 also colocalizes with aggregated tau in AD patients' brain lesions,^198^ suggesting its role in SG mediated tau aggregation. At the same time, it was found that acetylation decreases the SG‐association of tau.^125^ Because acetylated tau is associated with increased neurotoxicity, a protective role of SGs in the context of tau pathology might also be possible.

Stress granules consist of a “core”, which consists of highly concentrated RNA/protein components, and a “shell”. The shell, composed by less‐concentrated constituents, surrounds the core and possess weak interactions with it.^199^ Factors engaged in nucleocytoplasmic transport such as importins, exportins, and various nucleoporins (Nups) are stated to constitute the SG shells.^199^, ^200^ Eight of 30 different Nups contain intrinsically disordered regions in phenylalanine‐glycine repeats (FG).^201^, ^202^, ^203^, ^204^ Phosphorylated tau is reported to colocalize and interact directly with the FG‐domain of Nup98 in the nuclear membrane affecting its function and leading to impairment of nucleocytoplasmic transport.^205^ Given that tau aggregation is promoted by polyanionic macromolecules,^206^, ^207^, ^208^ the aggregation of tau was studied in the presence of the highly negatively charged free Nup98 C‐terminal part, which is typically located within the nucleopore complex.^205^ The data suggested that Nup98 can promote the fibril assembly of tau in vitro and that the accumulation of Nup98 in neurons potentially induces aggregation of tau.^205^

### 4.3 Liquid‐to‐solid phase transition

The aggregation pathways of tau from a soluble intrinsically disordered state into neurofibrillary tangles in AD is largely unknown and believed to follow typical sigmoidal kinetics. Soluble tau monomers can phase separate in cells and in vitro experiments suggest that condensates of tau serve as the precursors for the tau aggregates. However, it is still unclear how modulations of the conformational ensemble of monomeric tau come into play, and how or whether the phase‐separated tau droplets affect the assembly of tau into aggregates.

Phase separation of tau K18 slightly increases the β‐structure content along with the tendency to acquire β‐hairpin conformation as revealed by secondary structural analysis using CD and NMR.^120^, ^128^ Although tau condensates eventually become thioflavin T positive over incubation, the fluorescence intensity of the dye is much frailer as compared to what is detected in the presence of heparin,^120^, ^121^ suggesting a significantly smaller fraction of β‐structure content in the tau condensates as compared with that in the amyloid fibrils.

Whether the LLPS of tau is linked to fibrillization remains contentious. Factors that augment the aggregation of tau such as heparin, mutations, or hyperphosphorylation, can favor condensation of tau (see above). Interaction of protein disulfide isomerase with tau results in suppression of tau droplet formation as well as aggregation.^165^ Concurrently the already mentioned EFhd2, is identified as a tau‐associated protein in AD brains.^163^, ^209^ Recent studies demonstrate that EFhd2, in the presence of calcium, phase separates together with tau into liquid droplets suggesting that EFhd2 regulates liquid–liquid phase separation of tau.^164^ However, while EFhd2 has been shown to promote the formation of tau amyloid fibrils,^210^ it is uncertain whether it can regulate the process of tau aggregation. Moreover, a rapid transition of phosphorylated tau from the condensed phase to a gel‐like state was reported.^123^ In addition, Boyko et al. investigated the impact of disease‐related mutations on PEG‐induced tau LLPS and compared it to heparin‐induced fibrillization.^127^ The data suggested that, although none of the tested mutations influenced the phase separation propensity of htau40, LLPS does accelerate the formation of fibrillar aggregates, and this effect is especially dramatic for htau40 variants with disease‐related mutations.^127^

In contrast to PEG‐induced LLPS of htau40,^127^ self‐coacervation of K18^120^ as well as Hofmeister‐salt‐induced LLPS of Δtau187,^140^ RNA‐mediated LLPS of Δtau187 is partially independent of tau amyloid aggregation even though occurring in overlapping conditions.^141^ Besides, disease‐associated tau mutants can under certain conditions enhance tau phase separation and mature into more solid‐like states, but this was suggested to preferentially promote non‐filamentous oligomerization.^180^ The data highlight that the connection between tau LLPS and fibrillization is complex, but might be strongest in conditions in which hydrophobic interactions contribute to tau LLPS. So far in vitro or cell‐based investigations have been carried out to study phase separation of tau. A direct link between tau condensates and neurodegeneration in vivo is still lacking. Therefore, the bridging between liquid phase condensation and liquid–solid transitions of tau, especially in vivo, remains elusive and requires further investigation.

Tau protein stabilizes the microtubular cytoskeleton in addition to dynamically regulating other physiological activities including synaptic plasticity, neurite outgrowth, and nucleocytoplasmic transport in axons.^211^ Various etiological factors root the basis of atypical tau hyperphosphorylation through multiple pathways including Aβ metabolism, dysregulation of phosphorylation/dephosphorylation process, and impairment of glucose metabolism in the brain.^212^ Hyperphosphorylated tau has enhanced potential to disengage from microtubules,^213^ self‐assemble, and aggregate.^214^ On this basis, we propose a model for the possible mechanisms involved in disease pathology highlighting the probable contribution of phase transition of tau **(**Figure 6). Under normal conditions, tau facilitates the nucleation of microtubule polymerization through phase separation with tubulin in liquid‐like droplets^38^ and intact nucleocytoplasmic transport enables the regular conveyance of RBPs and RNA across the nuclear membrane. In contrast, when tau undergoes abnormal hyperphosphorylation in a diseased condition, it might lead to several derailed processes including destabilization of the microtubule bundles, impaired microtubule polymerization, and disruption of nucleocytoplasmic transport.^205^, ^215^ The impairment of nucleocytoplasmic transport would probably enhance the accumulation of RBPs, Nups, and RNA within soma where hyperphosphorylated tau would also be accessible to interact. The interaction of tau with RNA, RBPs, and nucleoporins facilitates its phase separation and the formation of condensates having a high concentration of tau monomers.^205^ These droplets might undergo liquid–liquid to liquid–solid transition and possibly mediate the formation of pathogenic tau fibrils. Given that tau interacts with the components of stress granules that facilitate its phase separation, it is likely that such interactions might also lead to the formation of pathological stress granules mediated by LLPS. This remains a matter of debate whether stress granule formation stimulates tau fibrillization directly from its monomeric state^13^ or tau condensates lead to tau aggregation via stress granule formation. Despite wide‐ranging studies, the exact molecular basis of tau condensation and its role in disease pathogenesis remains to be an area of intensive research.

## 5 CONCLUSION AND PERSPECTIVES

The cellular activities of tau range from microtubule polymerization to neuronal growth and axonal transport. Many of these activities are governed by PTMs that alter the charge pattern, affect the cellular localization, and regulate the interactions with surrounding counterparts. Being amphiphilic, tau has a high propensity to undergo LLPS, and a growing body of research has shed light on the physiological significance of LLPS of tau (Figure 3). However, aberrant phase transitions and aggregation of tau are linked to neurodegenerative diseases (Figure 6). This review highlights the ongoing efforts to elucidate the biophysical mechanism of liquid phase condensation and liquid‐to‐solid phase transition, and fibrilization of tau. An amalgamation of various existing and emerging methodologies described here provided a wealth of information on a wide range of spatiotemporal resolutions. We believe that emerging NMR, single‐molecule, super‐resolution, cryoEM, soft matter physics, and molecular simulations in conjunction with molecular and cell biology techniques will allow us to unmask the critical drivers of tau phase transition and its association with cell physiology and neurodegenerative diseases. It is worth delving deep by extending these studies in cellular systems or by mimicking the environment in the presence of other kinases, regulatory factors, and the protein quality control machinery.^111^ With the rise in LLPS studies of granular architectures and bodies, it would be interesting to have a kaleidoscopic view of stress granule associations with tau monomers, toxic intermediate species, and its involvement in tauopathies during stress.^193^ Moreover, tau plays an important role in regulating nucleocytoplasmic transport by interacting with other neuronal partners, such as tubulin, actin, and nucleoporins via condensate formation. Alterations in tau can cause liquid condensate to gel‐like transition leading to impairment in nucleocytoplasmic transport and neurotoxicity. This further necessitates targeted therapeutic strategies that reverse or delay the liquid–liquid to the liquid–solid transition, thereby restoring the physiological function or regulating the gain‐in‐toxic‐function of tau.

## AUTHOR CONTRIBUTIONS

**Sandeep K. Rai:** Writing‐original draft; writing‐review & editing. **Adriana Savastano:** Writing‐original draft; writing‐review & editing. **Priyanka Singh:** Writing‐original draft; writing‐review & editing. **Samrat Mukhopadhyay:** Conceptualization; funding acquisition; project administration; supervision; writing‐original draft; writing‐review & editing. **Markus Zweckstetter:** Conceptualization; funding acquisition; project administration; supervision; writing‐original draft; writing‐review & editing.

## CONFLICT OF INTEREST

The authors declare no competing financial interests.
